# Modulation of NLRP3 Inflammasome through Formyl Peptide Receptor 1 (Fpr-1) Pathway as a New Therapeutic Target in Bronchiolitis Obliterans Syndrome

**DOI:** 10.3390/ijms21062144

**Published:** 2020-03-20

**Authors:** Ramona D’Amico, Roberta Fusco, Marika Cordaro, Rosalba Siracusa, Alessio Filippo Peritore, Enrico Gugliandolo, Rosalia Crupi, Maria Scuto, Salvatore Cuzzocrea, Rosanna Di Paola, Daniela Impellizzeri

**Affiliations:** 1 Department of Chemical, Biological, Pharmaceutical and Environmental Sciences, University of Messina, Viale Ferdinando Stagno D’Alcontres 31, 98166 Messina, Italy; rdamico@unime.it (R.D.); rfusco@unime.it (R.F.); cordarom@unime.it (M.C.); rsiracusa@unime.it (R.S.); aperitore@unime.it (A.F.P.); egugliandolo@unime.it (E.G.); dimpellizzeri@unime.it (D.I.); 2 Department of Veterinary Science, University of Messina, Viale Annunziata, 98168 Messina, Italy; rcrupi@unime.it; 3 Department of Biomedical and Biotechnological Sciences, University of Catania, 95131 Catania, Italy; mary-amir@hotmail.it; 4 Department of Pharmacological and Physiological Science, Saint Louis University School of Medicine, 1402 South Grand Blvd, St Louis, MO 63104, USA

**Keywords:** inflammasome, bronchiolitis obliterans syndrome, inflammation

## Abstract

Chronic rejection is the major leading cause of morbidity and mortality after lung transplantation. Bronchiolitis obliterans syndrome (BOS), a fibroproliferative disorder of the small airways, is the main manifestation of chronic lung allograft rejection. We investigated, using transgenic mice, the mechanisms through which the deficiency of IL-1β/IL-18, Casp-1, or Fpr-1 genes could be protective in an experimental model of BOS, induced in mice by allogeneic heterotopic tracheal transplantation. Fpr-1 KO mice showed a marked reduction in histological markers of BOS and of mast cell numbers compared to other groups. Molecular analyses indicated that the absence of the Fpr-1 gene was able to decrease NF-κB nuclear translocation and modulate NLRP3 inflammasome signaling and the mitogen-activated protein kinase (MAPK) pathway in a more significant way compared to other groups. Additionally, Fpr-1 gene deletion caused a reduction in resistance to the apoptosis, assessed by the TUNEL assay. Immunohistochemical analyses indicated changes in nitrotyrosine, PARP, VEGF, and TGF-β expression associated with the pathology, which were reduced in the absence of the Fpr1 gene more so than by the deletion of IL-1β/IL-18 and Casp-1. We underline the importance of the NLRP3 inflammasome and the pathogenic role of Fpr-1 in experimental models of BOS, which is the result of the modulation of immune cell recruitment together with the modulation of local cellular activation, suggesting this gene as a new target in the control of the pathologic features of BOS.

## 1. Introduction

Chronic lung allograft rejection [1] is associated with an increased mortality rate and affects most lung transplant recipients within five years after transplantation [2]. Bronchiolitis obliterans syndrome (BOS) is characterized by chronic inflammation of the bronchial epithelium, which causes the infiltration of lymphocytes into the epithelial and subepithelial tissues and seriously damages normal epithelial cells [3,4]. Consequently, fibroblasts and myofibroblasts are induced to differentiation, which results in diffuse fibrosis, collagen and matrix deposition, and granulation tissue formation, ultimately leading to the occlusion of small airways [5,6]. Although BOS has been recognized for more than 60 years, little is known about its cellular and molecular pathogenesis. The therapeutic alternatives for BOS are limited and without a clearly established protocol. Possible treatments include changing immunosuppressive medication, azithromycin, plasmapheresis, and inhaled cyclosporine [7,8]. Although immunosuppression is increasingly being applied for the treatment of BOS, it is not enough to solve the problem of rejection. Thus, the development of a novel therapeutic approach for improving BOS-related mortality is needed. The innate immune system, with macrophages and neutrophils, plays a key role in the induction of the inflammatory process through the releasing of proangiogenic factors and inflammatory cytokines. Inflammasomes are a citoplamatic multiprotein complex found in macrophages, monocytes, and neutrophils, and are the main intracellular inflammatory pathways of the innate immune system [9]. Inflammasomes have also been found in the epithelial cells of tissues and mucosal surfaces. Five inflammasomes have been identified; of these, NLR Family Pyrin Domain Containing 3 (NLRP3) is the most characterized. This complex contains NLRP3, a NOD-like receptor that is a sensor for the activation of the inflammasome, and an apoptosis-associated speck-like protein containing a CARD complex (ASC), which binds pro-caspase through its CARD domain [10]. Pro-caspase is then activated in caspase-1 (Casp-1), which is a protease involved in cell apoptosis and regulates the inflammatory response through the release of cytokines [11]. In fact, Casp-1 is the main enzyme implicated in the cleavage of pro-interleukin (IL)-1β and pro-IL-18 into the biologically active cytokine [12]. Both cytokines drive an extensive range of pro-inflammatory networks in many cell types using common signal transduction cascades [13].

Moving upstream in the inflammatory cascade, formyl peptide receptors (FPRs) are found. Formyl peptide receptors (FPRs) are G protein-coupled receptors, whose main function is to sense the presence of harmful, pathogen-associated molecules or endogenous ligands, including classical biomarkers of inflammation and immune activation. In humans, the FPR family is constituted by three functional receptors (FPR1, FPR2, and FPR3) encoded by three genes, while in rodents, there are three genes encoding functional receptors and six genes encoding orphan receptors [14,15]. Fpr-1 is expressed in several phagocytic cell types, such as macrophages, monocytes, and neutrophils [16]. These cell types induce neutrophils and migrate into the lesion site through the activation of the FPRs. Inflammatory cells expressing FPRs, once they are recruited at the lesion site, are activated and trigger multiple pathways, e.g., increasing gene transcription, the assembly of intracellular pro-inflammatory complexes, and the release of reactive oxygen species and nitric oxide [14].

Moreover, it has recently been demonstrated that deletion of the Fpr-1 gene reduced tissue injury and inflammation in several experimental models, including those of endometriosis, colitis, and depression [17,18,19]. Considering that BOS is a disease of an inflammatory nature, which limits the survival rates of lung transplantation and other therapeutic options, we prepared an experimental study to investigate the cellular and molecular mechanisms involved in airway epithelial repair and regeneration [20,21]. The most used experimental model to study BOS (chronic rejection) is allogeneic, heterotopic tracheal transplantation in mice [22,23], which induces pathological changes similar to those seen in BOS. Therefore, the objective of this research focuses on the potential of two different but converging inflammatory pathways in therapeutic intervention for the treatment of BOS using transgenic mice.

## 2. Results

### 2.1. Histopatology Evaluation and Mast Cell Density in IL-1β/IL-18 KO, Casp-1 KO, and Fpr-1 KO

**Figure 1:**
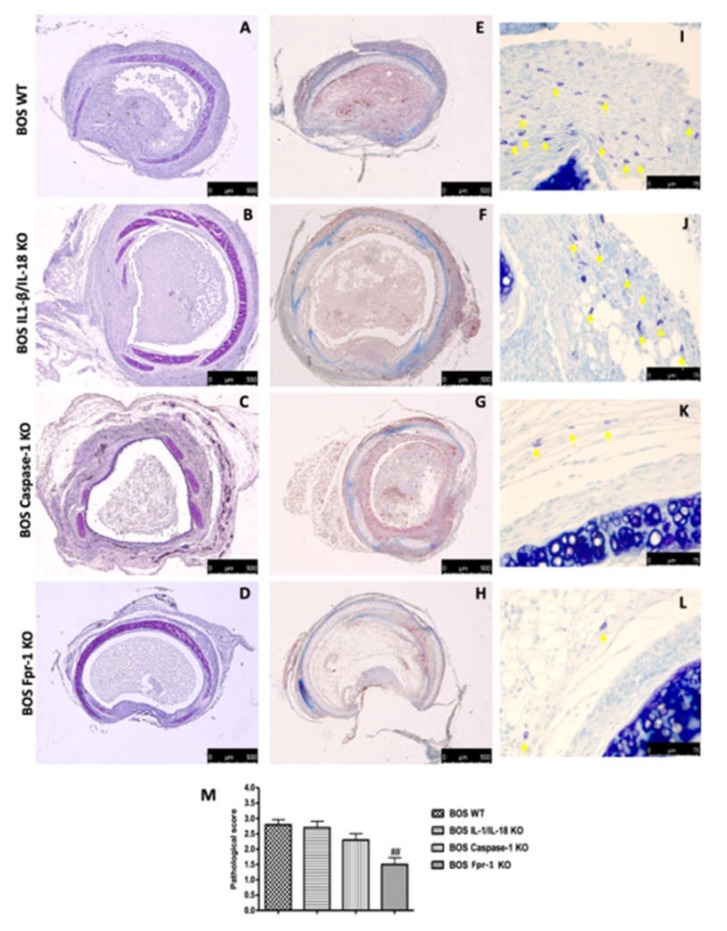
Histopatology evaluation and mast cell density in IL-1β/IL-18 KO, Casp-1 KO, and Fpr-1 KO: Histological evaluation of tracheal transplantation: wild-type (WT) (**A**), IL-1β/IL-18 KO (**B**), Casp-1 KO (**C**), Fpr-1 KO (**D**). Masson trichrome staining of the graft: WT (**E**), IL-1β/IL-18 KO (**F**), Casp-1 KO (**G**), Fpr-1 KO (**H**). Evaluation of mast cell degranulation by toluidine blue: WT (**I**), IL-1β/IL-18 KO (**J**), Casp-1 KO (**K**), Fpr-1 KO (**L**). Histopathologic score (**M**). For histological analyses, *n* = 5 animals from each group were employed. A *p*-value less than 0.05 was considered significant. ## *p* < 0.01 versus the WT group.

Histopathologic analysis of tracheal allografts from the Fpr-1 KO (Figure 1D) animals demonstrated marked reductions of the histological markers of BOS, such as ECM deposition, airway obliteration, loss of epithelial cell integrity, and leukocyte infiltration, compared to the wild-type (WT) group (Figure 1A). IL-1β/IL-18 KO (Figure 1B) and Casp-1 KO (Figure 1C) groups did not show significant differences from the WT group. Masson trichrome stain displayed that collagen deposition was very abundant in the WT animals (Figure 1E); similarly, the presence of collagen deposits was observed in IL-1β/IL-18 KO (Figure 1F) and Casp-1 KO (Figure 1G) groups. Sections taken from the Fpr-1 KO group showed a marked reduction in collagen deposition (Figure 1H). Mast cells numbers were enumerated via staining with toluidine blue. Compared to the WT group (Figure 1I), mast cell numbers in the Fpr-1 KO group were significantly reduced (Figure 1L). IL-1β/IL-18 KO (Figure 1J) and Casp-1 KO (Figure 1K) groups did not show significant differences from the WT group. The histopathologic score was significantly decreased in the tracheal allografts taken from Fpr-1 compared to other groups (Figure 1M).

### 2.2. Effects of the Absence of IL-1β/IL-18, Casp-1, and Fpr-1 on the NLRP3 Inflammasome Pathway

**Figure 2:**
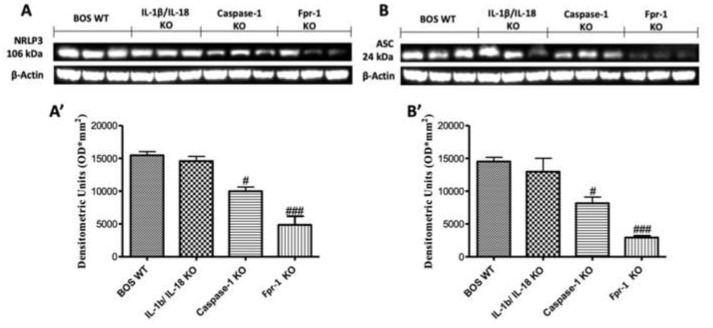
Effects of the absence of IL-1β/IL-18, Casp-1, and Fpr-1 on the NRLP3 inflammasome pathway. Western blots and, respectively, the densitometric analysis of NRLP3 (**A**,**A’**) and ASC (**B**,**B’**). For Western blot analyses, *n* = 5 animals from each group were employed. A *p*-value less than 0.05 was considered significant. # *p* < 0.05 versus the WT group, ### *p* < 0.001 versus the WT group.

**Figure 3:**
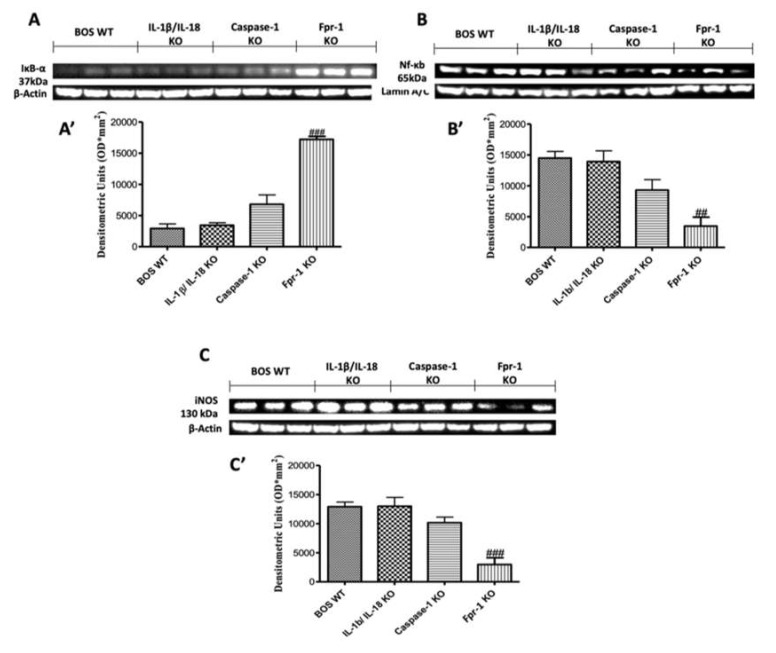
Effects of the absence of IL-1β/IL-18, Casp-1, and Fpr-1 on the NF-κB pathway and iNOS expression. Western blots and, respectively, the densitometric analysis of IkB-α (**A**,**A’**), NF-kB p65 (**B**,**B’**), and iNOS (**C**,**C’**). For Western blot analyses, *n* = 5 animals from each group were employed. A *p*-value less than 0.05 was considered significant. ## *p* < 0.01 versus the WT group, and ### *p* < 0.001 versus the WT group.

To better explore which signaling pathway could be involved in the inflammatory response on the BOS model, we performed Western blot analyses for the NLRP3 inflammasome pathway. The results obtained showed an important increase in NLRP3 expression in graft samples collected from the WT animals, while NLRP3 levels were significantly reduced in the Fpr-1 KO group (Figure 2A; Densitometric Analysis A’). The IL-1β/IL-18 KO mice showed results comparable to those observed in the WT group, while Casp-1 KO lightly attenuated the expression of NLRP3 (Figure 2A; Densitometric Analysis A’). Western blot analysis also displayed an upregulation of ASC levels in the WT group, which was significantly reduced in Fpr-1 KO mice (Figure 2B; Densitometric Analysis B’). Depletion of IL-1β and IL-18 genes did not show a reduction in ASC; in contrast, a low reduction in ASC expression was detected in the Casp-1 KO animals (Figure 2B; Densitometric Analysis B’). The NF-κB pathway is one of the most involved in the inflammation propagation. Western blot analysis showed a low IκB-α expression in samples from the WT mice compared to the Fpr-1 KO group (Figure 3A; Densitometric Analysis A’). In Casp-1 KO mice, a low reduction of IκB-α degradation was detected, but it was not significant, while absence of the IL-1β/IL-18 gene did not prevent IκB-α degradation (Figure 3A; Densitometric Analysis A’). Conversely, NF-κB levels in the nuclear fractions of samples were noticeably increased in WT and IL-1β/IL-18 KO animals 28 days after transplantation (Figure 3B; Densitometric Analysis B’). Absence of Casp-1 slightly decreased NF-κB expression, but the genetic deficiency of Fpr-1 decreased NF-κB expression in a more significant way (Figure 3B; Densitometric Analysis B’). Because NF-κB activation is linked to iNOS induction, iNOS expression was also evaluated via Western blot [24]. An increased expression of iNOS was found in WT mice as well as in the IL-1β/IL-18 KO group (Figure 3C; Densitometric Analysis C’). The absence of Casp-1 was not able to decrease iNOS expression in a significant way, while the genetic deficiency of Fpr-1 showed a greater effect compared to the other group (Figure 3C; Densitometric Analysis C’).

### 2.3. Effects of the Absence of IL-1β/IL-18, Casp-1, and Fpr-1 on Nitrotyrosine Formation and PARP Activation

**Figure 4:**
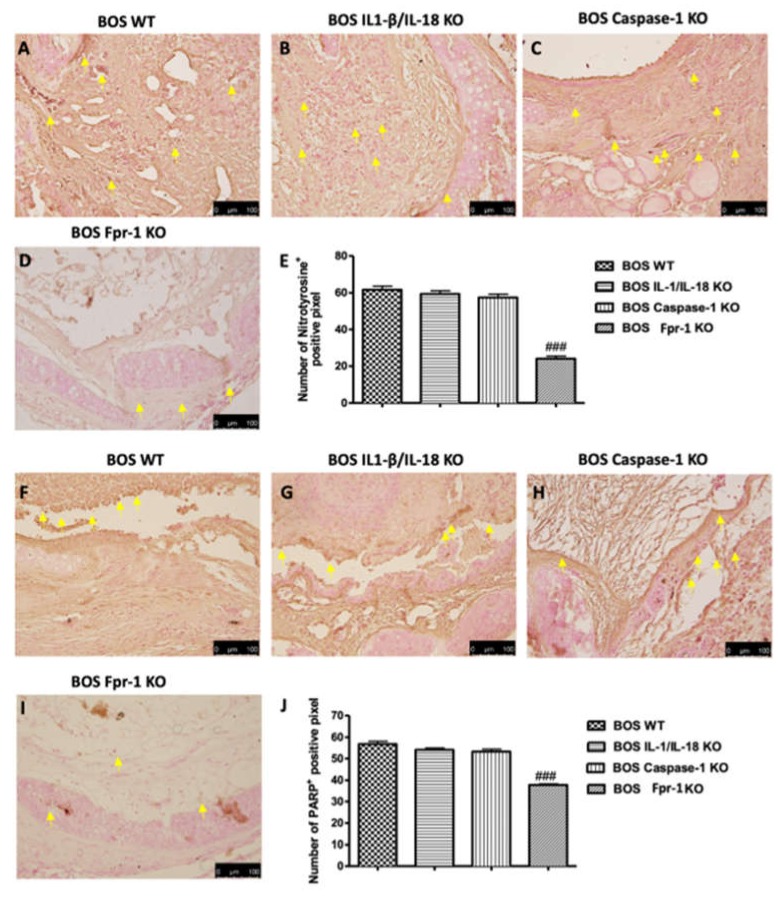
Effects of the absence of IL-1β/IL-18, Casp-1, and Fpr-1 on Nitrotyrosine formation and PARP activation. Immunohistochemistry evaluation of nitrotyrosine expression: WT (**A**), IL-1β/IL-18 KO (**B**), Casp-1 KO (**C**), Fpr-1 KO (**D**), densitometric analysis (**E**). Immunohistochemistry evaluation of PARP expression: WT (**F**), IL-1β/IL-18 KO (**G**), Casp-1 KO (**H**), Fpr-1 KO (**I**), densitometric analysis (**J**). Yellow arrows point the positive cells. For immunohistochemistry, *n* = 5 animals from each group were employed. A *p*-value less than 0.05 was considered significant. ### *p* < 0.001 versus the WT group.

To confirm the presence of nitrosative stress, immunohistochemical analysis of nitrotyrosine was performed. Tracheal transplantation sections obtained from the WT group displayed an important positive nitrotyrosine immunostaining (Figure 4A; Densitometric Analysis 4E); however, sections from Fpr-1 KO mice showed a significant reduction in the degree of nitrotyrosine immunoreactivity in the graft tissue (Figure 4D; Densitometric Analysis 4E). The IL-1β/IL-18 KO and Casp-1 KO groups did not supply a reduction in immunohistochemical staining (Figure 4B,C, respectively; Densitometric Analysis 4E). Additionally, we observed the expression of PARP, an indicator of DNA breakdown, via immunohistochemical analysis. A significant increase in positive staining for PARP was detected in graft tissues from the WT group (Figure 4F; Densitometric Analysis 4J); similarly, sections obtained from IL-1β/IL-18 KO and Casp-1 KO groups also showed a strong positive staining for PARP (Figure 4G,H, respectively; Densitometric Analysis 4J). Absence of Fpr-1 reduced the immunostaining of PARP in a significant manner (Figure 4I; Densitometric Analysis 4J).

### 2.4. Effects of the Absence of IL-1β/IL-18, Casp-1, and Fpr-1 on Apoptosis

**Figure 5:**
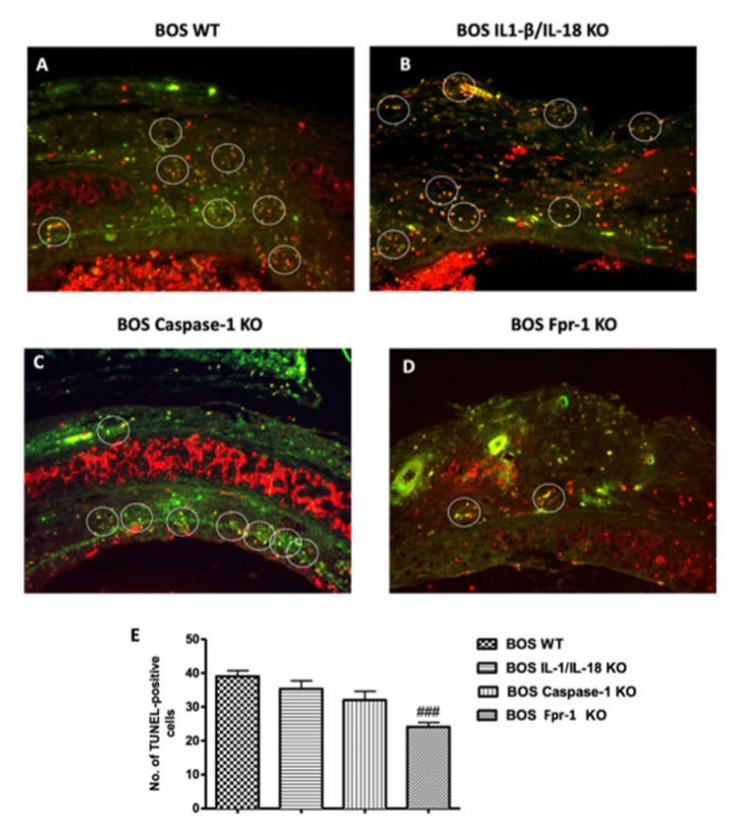
Effects of the absence of IL-1β/IL-18, Casp-1, and Fpr-1 on apoptosis. TUNEL staining of tracheal transplantation: WT (**A**), IL-1β/IL-18 KO (**B**), Casp-1 KO (**C**), Fpr-1 KO (**D**), graphical quantification (**E**). For TUNEL staining, *n* = 5 animals from each group were employed. A 20× magnification is shown (50-µm scale bar). A *p*-value less than 0.05 was considered significant. ### *p* < 0.001 versus the WT group.

To examine whether the apoptosis of airway epithelial cells is involved in the worsening of BOS, we performed the TUNEL assay on paraffin sections of allografts. TUNEL staining showed that there were more apoptotic cells in the grafts of WT mice (Figure 5A). The genetic deficiency of both IL-1β and IL-18 or Casp-1 showed a small decrease in the number of apoptotic cells, but this was not significant (Figure 5B,C, respectively). Absence of Fpr-1 showed a marked decrease in the number of apoptotic cells compared with the other groups (Figure 5D). The average number of TUNEL-positive epithelial cells per section was significantly decreased in the Fpr-1 KO group compared to other groups (Figure 5E).

### 2.5. Effects of the Absence of IL-1β/IL-18, Casp-1, and Fpr-1 on Grow Factors Expression

**Figure 6:**
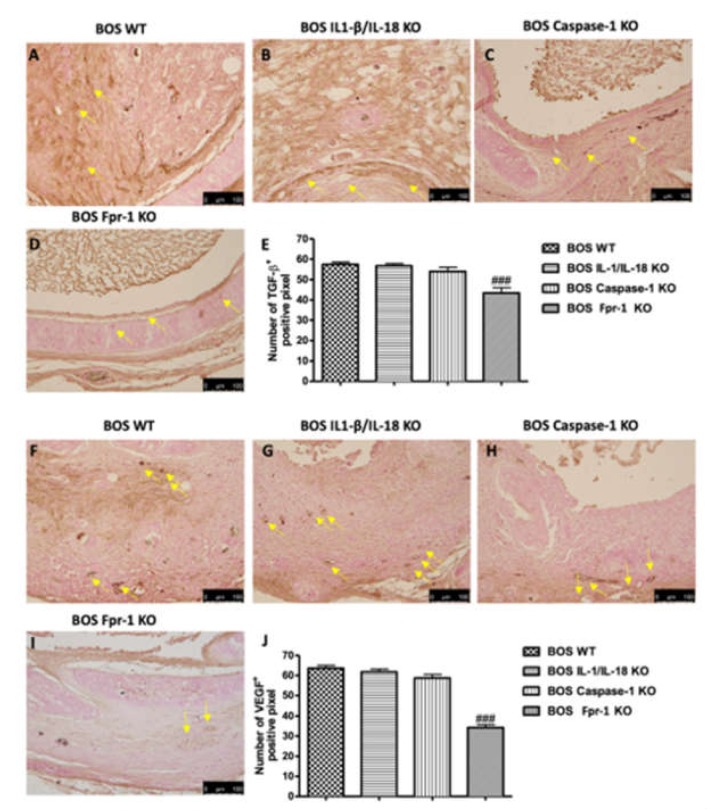
Effects of the absence of IL-1β/IL-18, Casp-1, and Fpr-1 on growth factor expression. Immunohistochemistry evaluation of VEGF expression: WT (**A**), IL-1β/IL-18 KO (**B**), Casp-1 KO (**C**), Fpr-1 KO (**D**), densitometric analysis (**E**). Immunohistochemistry evaluation of TGF-β expression: WT (**F**), IL-1β/IL-18 KO (**G**), Casp-1 KO (**H**), Fpr-1 KO (**I**), densitometric analysis (**J**). Yellow arrows point the positive cells. For immunohistochemistry, *n* = 5 animals from each group were employed. A *p*-value less than 0.05 was considered significant. ### *p* < 0.001 versus the WT group.

To help better delineate the underlying mechanisms of airway obstruction in the BOS model, we next investigated the expression of the growth factors, such as VEGF and TGF-β, by immunohistochemical staining. Since VEGF has been shown to be an important angiogenic factor, we assessed whether it contributes to the angiogenic activity in BOS. Twenty-eight days after transplantation, immunohistochemical analysis of the grafts displayed increased staining for VEGF in WT animals (Figure 6A; Densitometric Analysis 6E), which was reduced in Fpr-1 KO mice (Figure 6D; Densitometric Analysis 6E), whereas IL-1β/IL-18 KO and Casp-1 KO groups did not show a reduction in VEGF expression (Figure 6B,C, respectively; Densitometric Analysis 6E). Additionally, we evaluated the expression of TGF-β, an essential mediator of the fibroproliferative response present in BOS. Positive staining for TGF-β was higher in tissues obtained from the WT, IL-1β/IL-18 KO, and Casp-1 KO animals (Figure 6F–H, respectively; Densitometric Analysis 6J), while Fpr-1 KO mice showed a lower extent of staining for TGF-β (Figure 6I; Densitometric Analysis 6J), compared to other groups.

### 2.6. Effects of the Absence of IL-1β/IL-18, Casp-1, and Fpr-1 on the Mitogen-Activated Protein Kinase (MAPK) Pathway

**Figure 7:**
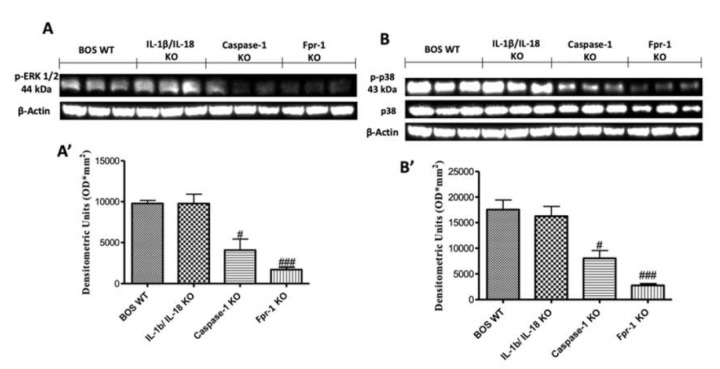
Effects of the absence of IL-1β/IL-18, Casp-1, and Fpr-1 on the mitogen-activated protein kinase (MAPK) pathway. Western blots and, respectively, the densitometric analysis of p-ERK 1/2 (**A**,**A’**) and p-p38 (**B**,**B’**). For Western blot analyses, *n* = 5 animals from each group were employed. A *p*-value less than 0.05 was considered significant. # *p* < 0.05 versus the WT group, ### *p* < 0.001 versus the WT group.

To investigate the cellular mechanisms by which the absence of IL-1β/IL-18, Casp-1, and Fpr-1 genes may attenuate the development of BOS inflammation, we also performed Western blot analyses for the mitogen-activated protein kinase (MAPK) pathway. The activation of MAPK pathways, in particular, the phosphorylation of ERKl/2 expression, was investigated by Western blot in graft tissues homogenates at 28 days after transplantation. A significant increase in pERK1/2 levels was observed in WT mice (Figure 7A; Densitometric Analysis A’). Absence of both IL-1β and IL-18 did not show a reduction in phosphorylation of ERKl/2 expression, while the genetic absence of Casp-1 significantly reduced p-ERK expression (Figure 7A; Densitometric Analysis A’). However, the genetic deletion of Fpr-1 offered a more protective action compared to the other groups (Figure 7A; Densitometric Analysis A’). Moreover, to confirm this data, we evaluated the phospho-p38 expression by Western blot analysis. A significant increase in the phospho-p38 expression was observed in WT and IL-1β/IL-18 mice (Figure 7B; Densitometric Analysis B’). Absence of Casp-1 reduced levels of p-p38 expression, but the deletion of Fpr-1 is better able to inhibit the increase of p-p38 expression compared to the other groups (Figure 7B; Densitometric Analysis B’).

## 3. Discussion

Currently, lung transplantation has become a principal alternative for patients with severe or terminal pulmonary diseases that cannot be cured completely by medical treatments [15]. Despite the considerable advances in immunosuppressive therapies in the care of lung transplant recipients, lung rejection is a common complication with an incidence that exceeds all other solid organ transplantations [25]. After three years, survival rates are between 50% and 70% [17,18]. Histologically, lung allograft rejection is seen as a progressive obliteration of small airways known as “bronchiolitis obliterans” (BO) and, consequently, BO-related syndrome (BOS). In detail, BOS is a chronic inflammatory process and is clinically manifested with the obstruction and obliteration of airways, characterized by an infiltration of peribronchiolar leukocytes that eventually invade and disrupt the submucosa, basement membrane, and airway epithelium [19]. This is followed by fibroproliferation, granulation tissue formation, and the accumulation of ECM, ultimately ending in fibro-obliteration of airways [20,21]. BOS has treatments with limited efficacy, and to understand the molecular mechanisms involved in the development of the disease, the murine experimental model of a heterotopic tracheal transplant is simple and quick for the study of new therapies. The present study using different transgenic mice aimed at a meticulous valuation of the inflammatory response in a mouse model of BOS. In this study, we compared the progression of BOS in IL-1β/IL-18 KO, Casp-1 KO, and Fpr-1 KO mice compared to WT animals. Our data demonstrate that the absence of Fpr-1 renders mice significantly less susceptible to the development of BOS compared to other groups. Such a strong outcome is reliant on a variety of cellular and tissue changes that seem under the control of this receptor, which is upstream in the inflammatory cascade.

In the present study, we showed that the absence of Fpr-1 was able not only to reduce histopathological marks of BOS, such as airway obliteration and the loss of epithelial cell integrity, but also to decrease collagen deposition. Moreover, the development of implants is associated with an increased number of intact and degranulated mast cells, as displayed in the WT group, while Fpr-1 KO mice showed a reduction in mast cell number in the tracheal transplantation compared to other transgenic groups. Additionally, we reported that the absence of Fpr-1 seems to increase the protective effects (ameliorated histological alteration and collagen deposition) because different converging inflammatory pathways are being inhibited. Firstly, it has been demonstrated that inflammasome pathways have high potential to be key player in some lung diseases, such as chronic inflammation and fibrosis response [22]. Inflammasomes are a group of cytosolic protein complexes implicated in the induction of innate immune/inflammatory response [23]. At present, there are five inflammasomes that are clearly identified: NLRP1, NLRP3, NLRC4, Pyrin, and AIM2 [26]; however, emerging evidence indicates that several other members of the NLR family and the PYHIN family, including NLRP6, NLRP7, NLRP12, and IFI16, can also form inflammasomes, but their composition remains obscure [27]. Among these inflammasomes, the NLRP3 inflammasome is the most studied because of its possible involvement in several human diseases. The exact mechanism of activation is still unclear, but new evidence suggests that a two-step mechanism activates NLRP3 [28]. A priming step results in the activation of the transcription factor NF-κB. This activation of NF-κB is critical for upregulating the transcription of both pro-IL-1β and NLRP3 itself. At this stage, the NLRP3 is in a signalling-incompetent conformation, and basal levels of NLRP3 are inadequate for efficient inflammasome formation. A second signal, provided by diverse agonists, promotes indirect activation of the inflammasome, by ion, ROS, or ATP [29]. In light of the above, we focused our attention on the NLRP3 inflammasome because it was previously demonstrated that its excessive activity contributes to acute and chronic allograft rejection [30]. In this study, the activation of inflammasome complex was observed 28 days after transplantation by Western blot analysis, as demonstrated by high expression of NLRP3 and ASC in samples taken from the WT mice, while Fpr-1 gene deletion led to a reduced activation of all the members of the NLRP3 inflammatory complex, compared to other groups where there was a small and not significant reduction. NF-kB is a chief regulator of inflammation, it manages several cellular processes such as apoptosis, cell proliferation, the secretion of cytokines, and oxidative stress [31]. In a normal condition, it is bound by the inhibitor protein IkB-α, which sequestered it into the cytoplasm. After the application of external stimuli, IkB-α is degraded, releasing NF-κB from the complex and allowing migration into the nucleus, where it activates the transcription of target genes. Grafts collected from Fpr-1 KO mice showed reduced IkB-α expression into the cytoplasm and NF-κB expression into the nucleus induced by the injury, compared to other groups. The inflammatory process generates ROS and nitrogen species, which provoke oxidative and nitrosative stress [32] and activate NADPH oxidase to generate significant, sometimes toxic, amounts of ROS (initially O_2_−), which propagate their signals that activate transcription factors. ROS can cause DNA damage, leading to poly ADP ribose synthase activation and cell death [33]. Therefore, the downregulation of iNOS expression can enhance the oxidative stress associated with tissue damage [24]. Indeed, in this study, Fpr-1 KO animals displayed reduced iNOS expression compared to the other transgenic mice. Moreover, we observed an increase of positive immunostaining for nitrotyrosine and PARP in WT mice. Deletion of Fpr-1 significantly reduced the expression of these markers, which may play a protective role against the damage of BOS. It is well known that apoptosis in the transplanted tissues or organs is a common phenomenon that affects the functional activity of the grafts. Previous studies have demonstrated the contribution of apoptosis to the damage of epithelial cells during the progression of BOS [34,35]. In this study, the apoptotic cells were readily observed within the transplanted tracheal epithelium cell and cartilage in the WT group. The genetic deficiency of both IL-1β and IL-18 or Casp-1 showed a small decrease in the percentages of apoptotic cells, and this was not significant. However, in Fpr-1 KO animals, TUNEL staining showed that the number of apoptotic cells decreased significantly 28 days after tracheal transplantation. Mast cells are also important for neo-angiogenesis [36], which guarantees oxygen supply to implants. Angiogenesis has also been found to be central to the progression of various chronic inflammatory pathologic conditions, including diabetic retinopathy, macular degeneration, pulmonary fibrosis, and RA [37,38]. These diseases are characterized by chronic inflammation and fibroproliferation associated with marked vascular remodeling. Since the fibro-obliteration process occurring during BOS is analogous to these fibroproliferative diseases, we hypothesized that vascular remodeling due to aberrant angiogenesis during fibro-obliteration of the allograft airway may contribute to the pathogenesis of BOS. VEGF is an angiogenic factor that promotes angiogenesis and neovascularization in tissue. We observed that a reduction in mast cell number caused by Fpr-1 gene deletion also led to a downregulation of VEGF, which is normally released by mast cells during inflammation. As mentioned above, the material obstructing the airway consists largely of collagen. This response is likely mediated in large part by a rise in the expression of TGF-β in the lumen and its surrounding cells. This suggests an important role for TGF-β in the tissue-remodeling response that is characteristic of transplant-associated BOS [39]. In fact, TGF-β stimulates collagen and fibronectin production in fibroblasts [40]; on the other hand, it can suppress the production of proteases that degrade the extracellular matrix [41]. Its expression is affected by the translocation of the transcription factor NF-kB from the cytoplasm to the nucleus [42]. This data is well in line with our results; in fact, we observed that the absence of Fpr-1 gene ameliorated the development of airway obstruction, more so than in other transgenic mice, through the downregulation of TGF-β expression. Moreover, TGF-β is known to induce its own gene expression through the Ras/MAPK signaling pathway [43]. Since the MAPK family of signal transducers has previously been implicated in promoting the transformation of fibroblasts into myofibroblasts in other preparations [44,45], we determined whether p38 or the extracellular signal-related (ERK) MAPKs could be activated in BOS. In this study, we found that the absence of the Fpr-1 gene significantly decreased the expression of these signalling intermediates more so in other groups compared to the WT mice. In conclusion, our study demonstrates that the chronic inflammatory process associated with the progressive obliteration of small airways induces proinflammatory signal transduction pathways by modulating the intracellular formyl peptide receptor system. Our evidence, well in line with the literature, suggests that NLRP3 inflammasome activation may also be involved in chronic obstructive pulmonary disease. It is modulated on multiple levels, ranging from transcriptional control to post-translational protein modifications. Mice with a targeted deletion of Fpr1 are significantly less vulnerable to the pathologic features of the secondary damage associated with BOS and induced by the activation of the NLRP3 inflammasome compared with the control, while IL-1β/IL-18 and Casp-1 KO animals were less susceptible than the WT but more than the Fpr-1 KO. This suggests that these two different inflammatory pathways might be new therapeutic targets modulating the pathological characteristics of BOS.

## 4. Materials and Methods

### 4.1. Animals

IL-1β/IL-18 double KO mice were obtained from Arturo Zychlinski (Max Planck Institute, Berlin, Germany), while Casp-1 KO mice were obtained from The Jackson Laboratory (Bar Harbor Maine, USA). Fpr-1 KO mice on the C57BL/6 genetic background and C57BL/6 animals (male 25–30 g), used as WT controls, were acquired from Envigo (Milan, Italy) and located in a controlled environment and provided with standard rodent chow and water. The University of Messina Review Board for the care of animals approved the research (9 February 2017, 137/2017-pr). Animal care was in conformity with current legislation for the protection of animals used for scientific purposes (Directive 2010/63/EU).

### 4.2. Animal Model of Tracheal Transplantation

Tracheas were transplanted as previously described [46,47,48]. Briefly, the mice were euthanized, and the tracheae were removed from donor mice via an anterior middle incision. The resected trachea was immediately placed in ice-cold PBS with penicillin G sodium (100 U/mL) and streptomycin sulfate (100 μg/mL) (Life Technologies). The receptor was subjected to inhalation anesthesia with isoflurane in a titrated dose to reach analgesia with spontaneous breathing, and a 0.5 cm horizontal incision was made in the midline neck region, in the dorsal suprascapular area. After the recipient’s trachea was transacted, the donor trachea was sewn in with 10-0 nylon sutures, and the overlying skin was closed with 5-0 silk. The grafts were harvested 28 days after the transplantation.

### 4.3. Experimental Groups

Mice were randomly allocated into the following groups (*n* = 10):

- The BOS WT group: mice were subjected to tracheal transplantation as described above.
- The BOS IL-1β/IL-18 KO group: mice were subjected to tracheal transplantation as described above, as well as the WT group.
- The BOS Casp-1 KO group: mice were subjected to tracheal transplantation as described above, as well as the WT group.
- The BOS Fpr-1 KO group: mice were subjected to tracheal transplantation as described above, as well as the WT group.

Mice were sacrificed 28 days after transplantation. Animals were anaesthetized with isoflurane and the implants were collected to perform all the histology and biochemical studies.

### 4.4. Histopatology and Mast Cell Evaluation

Tissue sections (7 μm) were deparaffinised, stained with haematoxylin/eosin (H/E), Masson’s Trichrome, and toluidine blue, and studied using light microscopy connected to an imaging system (LEICA DM6 with software LEICA LAS X Navigator). The histopatological score was determined as previously described [49]. Identification of mast cells in tracheal segments was performed as described previously [50].

### 4.5. Western Blot Analysis of Cytosolic and Nuclear Extracts from Tracheal Tissue

Western blot analysis was performed as previously described [51]. Cytosolic and nuclear extracts were divided. Brain tissues from each mouse were suspended in an extraction’s buffer containing 0.15 µM pepstatin A, 0.2 mM phenylmethylsulfonyl fluoride (PMSF), 1 mM sodium orthovanadate, and 20 µM leupeptin, which was homogenized at the highest setting for 2 min and centrifuged at 1000× *g* for 10 min at 4 °C. Supernatants contain the cytosolic fractions, while the pellets represent the nuclear ones. Pellets were re-suspended in a second buffer containing 150 mM sodium chloride (NaCl), 1% Triton X-100, 1 mM ethylene glycol tetraacetic acid (EGTA), 10 mM tris–chloridric acid (HCl) pH 7.4, 0.2 mM PMSF, 1 mM Ethylenediaminetetraacetic acid (EDTA), 0.2 mM sodium orthovanadate, and 20 µm leupeptin. After centrifugation at 4 °C and 15,000× *g* for 30 min, the nuclear protein contained the supernatants were stored at −80 °C for further analysis. The following primary antibodies were used: anti-NLRP3 (1:500, Santa Cruz Biotechnology, Heidelberg, Germany), anti-ASC (1:500, Santa Cruz Biotechnology, Heidelberg, Germany, #sc22514R), anti-IκBα (1:500, Santa Cruz Biotechnology, Heidelberg, Germany, #sc1643), anti-NF-κB p65 (1:500, Santa Cruz Biotechnology, Heidelberg, Germany, #sc8008), anti-iNOS (inducible-Nitric oxide synthases; 1:500, BD transduction, San Jose, CA, USA), anti-pp38 (1:500, Cell Signaling, Heidelberg, Germany), and anti-p-ERK (1:500, Santa Cruz Biotechnology, Heidelberg, Germany, #sc7383) in 1 x PBS, 5% *w/v* nonfat dried milk, and 0.1% Tween-20 at 4 °C, overnight. To ensure that blots were loaded with equal amounts of proteins, they were also probed with antibody against b-actin protein (cytosolic fraction 1:500; Santa Cruz Biotechnology) or lamin A/C (nuclear fraction 1:500 Sigma–Aldrich Corp.). Signals were examined with enhanced chemiluminescence (ECL) detection system reagent according to the manufacturer’s instructions (Thermo, USA). The relative expression of the protein bands was quantified by densitometry with BIORAD ChemiDocTM XRS+software and standardized to b-actin and lamin A/C levels. The blot was stripped with glycine 2% and re-incubated several times to optimize detection of proteins and to visualize other proteins minimizing the number of gels and transfers.

### 4.6. Immunohistochemical Localization of Nitrotyrosine, Poly (ADP-Ribose) Polymerase (PARP), Vascular Endothelial Growth Factor (VEGF), and Transforming Growth Factor-Beta (TGF-β)

Immunohistochemical analysis was performed as previously described [36]. Tracheal tissues were fixed in 10% (*w/v*) PBS-buffered formaldehyde and embedded in paraffin. Seven micrometer sections were prepared from the samples. After deparaffinization, endogenous peroxidase was quenched with 0.3% (*v/v*) H2O2 in 60% (*v/v*) methanol for 30 min. The slides were permeabilized with 0.1% (*w/v*) Triton X-100 in PBS for 20 min. Non-specific adsorption was decreased by incubating the section in 2% (*v/v*) normal goat serum in PBS for 20 min. Endogenous avidin or biotin binding sites were blocked by sequential incubation for 15 min with commercial avidin and biotin (Vector Laboratories, Burlingame, CA, USA), respectively. Subsequently, the sections were incubated overnight with anti-Nitrotyrosine (1:250, Merck-360 Millipore), anti-PARP (1:200, Santa Cruz Biotechnology, #sc1561), anti-VEGF (1:200, Santa Cruz Biotechnology, #sc7269), and anti-TGF-β (1:250, Santa Cruz Biotechnology, #sc17792). Sections were washed with PBS and incubated with peroxidase-conjugated bovine anti-mouse IgG, secondary antibody (1:2000 Jackson Immuno Research, WestGrove, PA, USA). Specific labeling was provided with a biotin-conjugated goat anti-mouse IgG and avidin-biotin peroxidase complex (Vector Laboratories, Burlingame, CA, USA). Images were collected using a Zeiss microscope and Axio Vision software (Carl Zeiss, Rome, Italy). The digital images were opened in ImageJ (National Institutes of Health, Bethesda, MD, USA), followed by deconvolution using the color deconvolution plug-in. When the Immunohistochemistry (IHC) Profiler plugin is selected, it mechanically plots a histogram profile of the deconvoluted diaminobenzidine image, and a corresponding scoring log is exhibited [52]. The histogram profile relates to the positive pixel intensity value obtained from a computer program [53]. All immunohistochemical analyses were carried out by 2 observers blinded to the treatment.

### 4.7. Terminal Deoxynucleotidyl Nick-End Labeling (TUNEL) Assay

Grafts apoptosis was analyzed by terminal deoxynucleotidyl transferase dUTP nick end labeling assay (TUNEL) using an in situ cell death detection kit. A double-staining technique was used. TUNEL staining for apoptotic cell nuclei was performed as described previously [34].

### 4.8. Materials

Unless otherwise stated, all compounds were purchased from Sigma-Aldrich. All solutions used for in vivo infusions were prepared using nonpyrogenic saline (0.9% NaCl; Baxter Healthcare Ltd., Thetford, Norfolk, UK).

### 4.9. Statistical Evaluation

All values are expressed as mean ± standard error of the mean (SEM) of *n* observations. For in vivo studies, *n* represents the number of animals used. In experiments involving histology or immunohistochemistry, the figures shown are representative of the least 3 experiments performed on diverse experimental days on tissue sections collected from all animals in each group. The results were analyzed by one-way analysis of variance (ANOVA) followed by a Bonferroni post-hoc test for multiple comparisons. A *p*-value less than 0.05 was considered significant. # *p* < 0.05 versus the WT group, ## *p* < 0.01 versus the WT group, and ### *p* < 0.001 versus the WT group.

## Abbreviations

ASC	apoptosis-associated speck-like protein containing a CARD complex
BOS	bronchiolitis Obliterans Syndrome
DAG	diacylglycerol
Casp-1	Caspase-1
FPR1	n-formylpeptide receptor 1
GDP	guanosine diphosphate
GPCR	G protein-coupled receptor
GTP	guanosine triphosphate
IL-1β	interleukin 1β
IL-18	interleukin 18
iNOS	inducible nitric oxide synthase
IP3	1,4,5-trisphosphate
MPO	myeloperoxidase
NF-κB	nuclear factor kappa B
NLRP3	NLR Family Pyrin Domain Containing 3
PBS	Phosphate buffered saline
PIP2	5-bisphosphate
PKC	protein kinase C
PLCβ	phospholipase C β
PMN	polymorphonuclear leukocyte
PMSF	phenyl-methyl sulfonyl fluoride
ROS	reactive oxygen species
SDS	sodium dodecyl sulphate
